# The Inhaler Technique Questionnaire (InTeQ): Development and Validation of a Brief Patient-Reported Measure

**DOI:** 10.3390/ijerph19052591

**Published:** 2022-02-23

**Authors:** Catalina Lizano-Barrantes, Olatz Garin, Alexandra L. Dima, Eric van Ganse, Marijn de Bruin, Manon Belhassen, Karina Mayoral, Àngels Pont, Montse Ferrer

**Affiliations:** 1 Health Services Research Group, IMIM-Hospital del Mar Medical Research Institute, 08003 Barcelona, Spain; clizano@imim.es (C.L.-B.); ogarin@imim.es (O.G.); kmayoral@imim.es (K.M.); apont@imim.es (À.P.); mferrer@imim.es (M.F.); 2 Department of Experimental and Health Sciences, Universitat Pompeu Fabra, 08003 Barcelona, Spain; 3 Department of Pharmaceutical Care and Clinical Pharmacy, Faculty of Pharmacy, Universidad de Costa Rica, San Jose 2060, Costa Rica; 4 Centro de Investigación Biomédica en Red de Epidemiología y Salud Pública CIBERESP, 28029 Madrid, Spain; 5 Research and Development Unit, Institut de Recerca Sant Joan de Déu, 08830 Barcelona, Spain; 6 Research on Healthcare Performance (RESHAPE), INSERM U1290, Université Claude Bernard Lyon 1, 69003 Lyon, France; eric.van-ganse@univ-lyon1.fr; 7 Respiratory Medicine, Croix-Rousse University Hospital, 69004 Lyon, France; 8 PELyon, Pharmacoepidemiology, 69007 Lyon, France; manon.belhassen@pelyon.fr; 9 Scientific Center for Quality of Healthcare (IQ Healthcare), Radboud Institute for Health Sciences, Radboud University Medical Center, 6500 HB Nijmegen, The Netherlands; marijn.debruin@radboudumc.nl; 10 Department of Pediatrics, Obstetrics and Gynaecology and Preventive Medicine, Universitat Autònoma de Barcelona, 08193 Barcelona, Spain

**Keywords:** asthma, inhalation technique, measurement instruments, patient-reported outcomes

## Abstract

There is a need for instruments designed for patients with asthma to self-report their performance of inhaling steps. We aimed to develop an accessible and easy-to-use patient-reported tool for inhaler technique assessment, which could also serve as a training and monitoring resource for any type of inhaler device, and to evaluate its feasibility, validity, and reliability in adults with asthma. The development was based on literature review and pilot testing with clinicians and patients. The Inhaler Technique Questionnaire (InTeQ) asks about the frequency of performing five steps when using inhalers (on a five-point Likert scale). We analyzed data from adults with persistent asthma (*n* = 361). We examined the measurement model using Mokken scaling analysis, construct validity by assessing hypotheses on expected discrimination among known groups, and reliability based on internal consistency and reproducibility. Means of the InTeQ items were in the range of 0.23–1.61, and coefficients of homogeneity were above the cutoff point, demonstrating the unidimensionality of the scale. Known groups’ global score differences were statistically significant between patients reporting having “Discussed in detail” or having “Not discussed/Only in general” the inhaler technique with their healthcare providers (*p* = 0.023). The Cronbach’s alpha coefficient was 0.716, and the intraclass correlation coefficient was 0.775. The InTeQ is a feasible, valid, and reliable instrument for self-reporting inhaler technique on any type of device.

## 1. Introduction

Asthma is a common chronic respiratory condition [1] which causes a substantial burden of disease [2]. Inhaled medications are the cornerstone of asthma management [1,3,4,5], but clinical evidence suggests that asthma control is often not achieved in practice. Poor inhaler technique is one of the main contributing factors [6], as it can result in suboptimal drug delivery and compromise treatment effectiveness [7,8,9,10]. Besides its difficult acquisition, inhaler technique deteriorates over time [11], with approximately 50% of patients failing to maintain it appropriately [12]. Therefore, it has to be assessed and improved repeatedly, particularly before considering any step-up in treatment when asthma remains uncontrolled [1].

Although the assessment of inhalation technique at every opportunity is recommended [1,13], it is rarely performed systematically with patients after their first prescription of inhaled medication [14,15]. Checklists are the most common, feasible, and accessible method with which to assess inhalation technique [1,9,16]. Most are intended for assessments performed by healthcare professionals [17] with considerable variation [6,9,16,18] in sources [18], steps included (from 3 to 21), and scoring systems [6,9,16].

We have found only two patient-reported questionnaires developed for inhaler technique assessment [19,20], both specifically for metered-dose inhalers. One focuses mainly on the steps for device use, with nine questions [19], and the other comprises steps for device preparation and use and for spacer use, with twenty questions [20]. However, most studies ask patients to self-assess their inhaler technique or how confident they are about appropriate use with one [21,22,23,24,25] to three questions [26]. To our knowledge, there are no instruments designed to self-report the performance of the inhaler technique steps common to any type of device.

Therefore, we aimed to develop a new patient-reported tool for inhaler technique assessment, the InTeQ (Inhaler Technique Questionnaire), which could also serve as a simple training and self-monitoring resource for any type of inhaler device. We aimed to evaluate the feasibility, validity, and reliability of the InTeQ in adults with asthma.

## 2. Materials and Methods

### 2.1. Development of the Inhaler Technique Questionnaire (InTeQ)

Our target was to design a brief assessment questionnaire for patients with asthma to measure the quality of their inhaler technique in their daily living. The development was based on a literature review of existing instruments and pilot testing with clinicians and patients. It was developed in English and translated in parallel into French by a specialized company using forward translation, independent back translation, review with an investigator’s input, and independent proofreading. The resulting English and French versions were pretested with 5 adult patients and 4 caregivers through cognitive interviews.

Although the ideal checklists are those standardized across devices as much as possible [9], and the metered-dose inhalers (MDI) have relatively similar recommendations for administration, the dry powder inhalers (DPI) have several designs and mechanisms for preparing the dose that slightly modify the administration requirements. Few common steps apply to both MDIs and DPIs, and there are some contrasting recommendations (e.g., shaking vigorously is important for MDIs, but detrimental for some DPIs). Checklists may include steps related to 3 overall processes: the preparation of the device/loading of the dose, the delivery of the dose, and the preparation of the device for storage [16]. For this questionnaire, we focused on key steps of the delivery of the dose process common across inhaler types.

Previous questionnaires [19,20] require patients to select the correct response option for each inhalation technique step. Choosing between focusing on correctness or on frequency was a major decision in the InTeQ design, which was finally based on information obtained in cognitive interviews. Table 1 shows the description of a patient-interviewee’s experience with using the inhalers, indicating that, even for a person who has received training, inhaler technique may vary. The patient has an overall perception that, at times, they may not be performing all steps at the optimum level. This suggests that inquiring about the frequency with which a patient performs the key steps of the inhaler technique (as opposed to whether they know or perform the various steps correctly or not) may be a useful approach.

We conceptualized the inhaler technique in this questionnaire as the frequency with which a patient appropriately performs key steps when administering inhaled medication. This approach is consistent with new electronic devices for measuring the timing of administration and the quality of technique at each inhalation, which have shown association with positive clinical outcomes [27,28]. Moreover, our approach is in line with methodological recommendations for health behavior change assessment and intervention [29].

In the final version of the InTeQ, respondents were asked to report on how often they performed five key steps when using their inhaler on a 5-point Likert scale from “Never” to “Always”, and a response option to indicate uncertainty (“Don’t know”; see Table 1). The InTeQ was revised and modified iteratively within the ASTRO-LAB project [30], with extensive pretesting until achieving this final version.

### 2.2. Study Design and Participants

The ASTRO-LAB project has been described elsewhere [30,31,32,33]. Briefly, it was a prospective observational study conducted in France and the United Kingdom, designed to provide new evidence about the safety of long-acting β-agonists in routine clinical care. Inclusion criteria were: aged 6–40 years, ≥6 months of prescribed use of controller inhalers during the 12 months before enrolment, no history of omalizumab therapy and/or any other concomitant respiratory disease, no chronic oral corticosteroid use during the 3 months before enrolment, and no asthma exacerbations 2 months before enrolment.

We analyzed data from adult patients (18–40 years old) from the ASTRO-LAB project, which was approved by the ethics and regulatory boards of the participating centers and was conducted following the Declaration of the World Medical Association. Written informed consent was obtained from all participants before inclusion.

### 2.3. Study Variables

Participants were followed through online surveys at 12-month intervals, with computer-assisted telephone interviews every 4 months and monthly text messages to detect severe asthma exacerbations. Demographic information and asthma severity markers were collected from the primary care records.

The yearly online survey included the InTeQ (inquiring about the previous 4 months), a question about the use of a spacer (“During the last 4 months, have you used your inhaler(s) with a spacer: always, often, sometimes, rarely, never or don’t know?”), and two questions related to the support that patients received from healthcare professionals: “Looking at the care you have received in the last year, please indicate how much this topic has been discussed with you: (A) Make together a concrete plan of where, when, and how I need to use my inhalers, and (B) Teach me how to use my inhaler.” These were answered with a numeric scale from 1 (‘not at all’) to 7 (‘in a lot of detail’).

Telephone interviews were used to collect patient-reported data on asthma treatment use and adherence with the Medication Intake Survey–Asthma [33], among others. The type of device for controller treatment was classified from manufacturers’ technical information into DPIs, MDIs, and breath-actuated MDIs (BA-MDIs).

### 2.4. Analytical Strategy

The information analyzed for this study was from the first online survey, except for the assessment of reproducibility, in which follow-up data until twelve months were also used. Patients’ characteristics were described by calculating percentages or means and standard deviations. The distribution of the InTeQ items was examined according to the reported use of reliever treatment, types of inhaler devices, and countries, and feasibility was evaluated through completion rate and data completeness.

We examined the InTeQ measurement model using Mokken scaling analysis (MSA), which assesses whether an item set orders respondents accurately on a continuum representing a latent trait [34]. We estimated the statistics of central tendency and dispersion of the items [35], inter-item correlations to identify any negative correlations, and multivariate outliers (Mahalanobis D^2^ *p* < 0.001). Finally, unidimensionality was tested by examining homogeneity and by performing an automated selection procedure [36] of items dichotomized into “Always” vs. the rest, due to their skewed distribution. Loevinger’s homogeneity coefficient (*H_i_* between each item and the item set, and *H* between all items) thresholds are: 0.3–0.4 (weak), 0.4–0.5 (medium), and 0.5–1 (good). After confirming unidimensionality, a global score was defined as a variable that counts the number of the InTeQ items answered as “Always”, ranging from 0 to 5 (the best inhaler technique). This global score orders patients along a continuum from systematically performing all 5 steps through to less frequent rigorous inhaler technique.

We evaluated construct validity by assessing the ability of the InTeQ items and its global score to discriminate among known groups defined by the support received from healthcare practitioners (inhaler technique “Not discussed/only in general” vs. “Discussed in detail”) and by spacer use with MDI (“Always–Often–Sometimes” vs. “Rarely–Never”). The hypotheses raised a priori were: patients discussing in detail with healthcare professionals and those with MDI using a spacer “Rarely–Never” might have better inhaler technique. To assess the discrimination capacity of the InTeQ items, the five response options were collapsed into three categories (Always, Often–Sometimes, and Rarely–Never); and the InTeQ global score was categorized into: 4–5 (Good inhaler technique), 3 (Fair), and 1–2 (Poor). Differences were tested using Chi-square.

Internal consistency was estimated in the total sample with Cronbach’s alpha coefficient. To evaluate reproducibility, we estimated the agreement between baseline and the 12-month follow-up in the subsample of stable participants (those reporting no exacerbations between both responses) with the kappa coefficient at item level and with the intraclass correlation coefficient (ICC) for the InTeQ global score. The difference between baseline and 12-month follow-up on the InTeQ global score was tested using paired Wilcoxon signed ranks test.

The prevalence of correct inhaler technique was displayed using an alluvial plot, an infographic that allows the representation of multiple pathways.

Of the 388 participants answering the online survey, 361 (93%) completed the InTeQ. Considering these and a Type I error of 0.05, the statistical power was 0.8 [37] to detect differences between known groups of 15% in the InTeQ items, with a kappa of 0.5 (SE 0.077) and a 95%CI of +/−0.15 in the subsample of stable participants.

## 3. Results

Around 60% of the participants were women, and 75% were living in France (Table 2). Almost all declared no hospitalizations in the 12 months before (96.6%), 71% used inhaled corticosteroids and long-acting β-agonists in fixed-dose combinations, and 64% used DPIs. Most participants (81.6%) never used a spacer, and very few (14%) reported having made a detailed inhaler plan.

**Figure 1:**
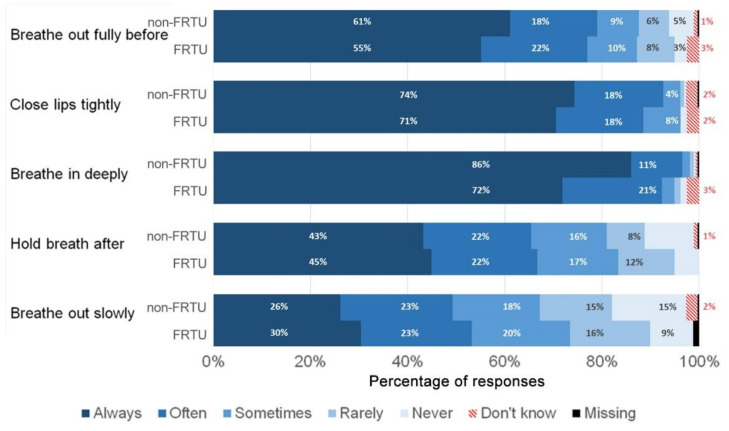
Distribution of InTeQ items (frequency of performing step during the last 4 months). FRTU: frequent reliever treatment use; non-FRTU: non-frequent reliever treatment use.

Figure 1 shows that the frequency of items responded with “Always” in patients with frequent and non-frequent reliever treatment use (FRTU and non-FRTU) varied from 30% and 26% (‘Breathe out slowly’) to 72% and 86% (‘Breathe in deeply’). For all items, less than 3% of participants answered “Don’t know”, and the proportion of missing items was less than 1%. The comparison of response distributions showed no statistically significant differences between FRTU and non-FRTU.

**Figure 2:**
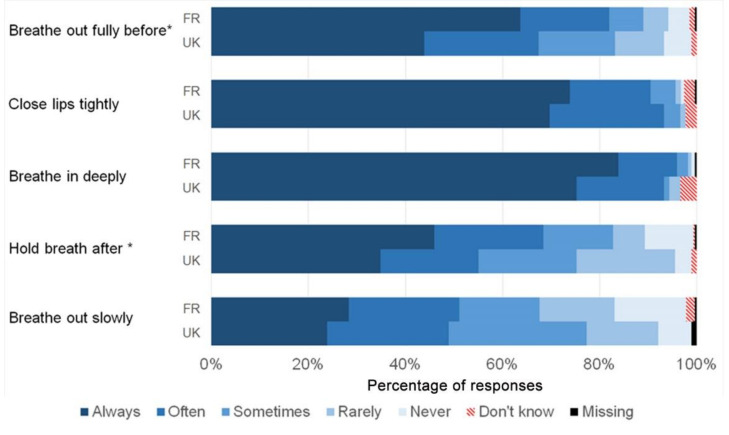
Comparison of inhalation technique between French and British participants. *: significant at *p* < 0.05 (0.008 and 0.001, respectively).

**Figure 3:**
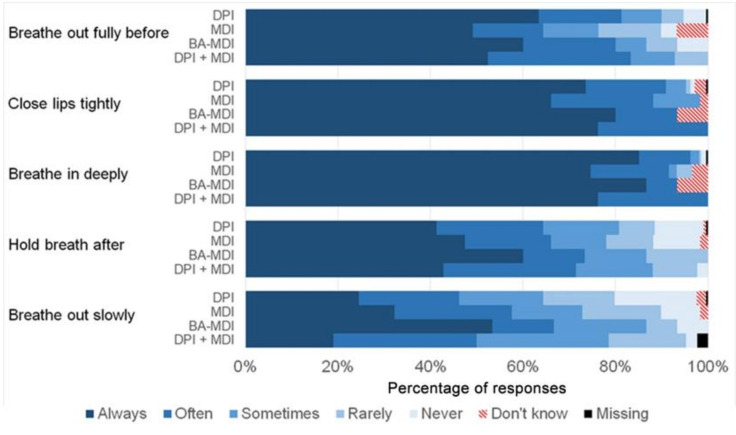
Comparison of inhalation technique between inhaler device types. DPI: dry powder inhaler; MDI: metered-dose inhaler; BA-MDI: breath-actuated metered-dose inhaler; DPI + MDI: more than one type of device.

French patients answered “Always” more frequently (Figure 2), with statistically significant differences for ‘Breathe out fully before’ and ‘Hold breath after’ (*p* = 0.006 and 0.001, respectively). The comparison of response distributions among inhaler device types (Figure 3) showed no statistically significant differences.

Table 3 shows that the five-point Likert response scale of the InTeQ items was skewed, with means in the range of 0.23–1.61 and inter-item correlations of 0.20–0.58. The homogeneity of all the dichotomized InTeQ items presented *H*_i_ and the summary *H* coefficient of the scale (*H* = 0.607) above the cutoff point of 0.3. An exploration of the scale’s unidimensionality with increasing homogeneity thresholds via an automated item selection procedure indicated that, at homogeneity threshold levels 0.30 to 0.50, all items belonged to the same scale.

Table 4 shows the results of the InTeQ’s validity based on the known groups. Statistically significant differences between the groups defined according to “Make an inhaler use plan together” were observed in only one InTeQ item (*p* = 0.049) and in the global score (*p* = 0.023). Patients that reported having “Discussed in detail” their inhaler technique with healthcare professionals answered more frequently having followed the steps “Always” than those who reported “Not discussed/only in general”. Additionally, one InTeQ item presented a statistically significant difference on spacer use among patients with MDI (*p* = 0.035).

The Cronbach’s alpha coefficient estimated in the total sample was 0.716. Table 5 shows the test–retest reproducibility results in the stable subsample. In total, 105 participants answered the InTeQ twice (at baseline and 12 months) and, after excluding 24 who suffered exacerbations, 81 patients were included in this subsample. Agreement ranged from 75.7% to 81.1%, and the kappa coefficient was moderate for four of the items (0.460–0.549). Global score change was not statistically significant and the intraclass correlation coefficient was 0.775.

**Figure 4:**
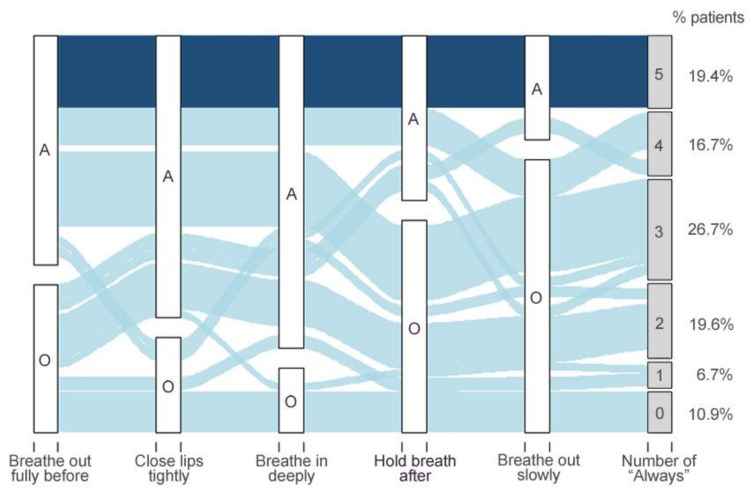
Alluvial plot showing the prevalence of correct inhaler technique. White vertical bars correspond to each InTeQ item; “A” indicates patients responding “Always”; “O” indicates patients responding other options (“Often–Sometime–Rarely–Never”). Gray bars on the right correspond to the number of items to which patients responded “Always”: the lowest horizontal band is patients that did not respond “Always” to any item, while the top, dark-blue band is patients who responded “Always” to every item. Width of the horizontal bands is proportional to the flow of patients’ responses.

Figure 4 shows the prevalence of correct inhaler technique: 19.4% of patients reported “Always” performing the five steps and 10.9% did not report “Always” in any step.

## 4. Discussion

To the best of our knowledge, this is the first study describing the development and metric properties of an instrument for self-reporting the frequency of adequately performing the key common steps of inhaler technique for any type of device. With the InTeQ, it was possible to estimate the actual prevalence of correct inhaler technique in real life. The InTeQ showed good feasibility, based on the high response rate and negligible missing data; good unidimensionality, which allows us to calculate a global score; good construct validity, based on the capacity of discriminating among known groups; and good reliability, based on internal consistency and test–retest reproducibility.

The selection of items for the InTeQ considered the five most important steps in the dose delivery process of the inhaler technique that were generic across devices. Four of the steps composing the InTeQ were among the five most common errors in inhaler use identified in a systematic review [6], and there is scientific evidence [38,39,40] supporting that the five steps selected for this review are the most common errors in inhaler use. The abovementioned systematic review reports ‘no post-inhalation breath-hold’ as the most frequent error for both types of devices, and 31% for overall prevalence of poor inhaler technique, both results that are consistent with our findings. Furthermore, the three InTeQ steps where more patients did not answer “Always” (‘Breathe out slowly’, ‘Hold breath after’, and ‘Breathe out fully before’) were among the 12 steps generic for all devices in the CRITIKAL study [41].

The CRITIKAL study showed association with uncontrolled asthma for four and three steps of the InTeQ in DPIs and MDIs, respectively [41], since ‘Breathe in deeply’ did not in MDIs. Nonetheless, this step has been linked to an improvement in quality of life [42]. It has to be noted that ‘Breathe in deeply’ involves insufficient respiratory effort for DPIs, and slow inhalation for MDIs. Finally, even though ‘Breathe out after’ was not related to uncontrolled asthma or exacerbations, it was among the most frequent errors in all devices [41]. Other inhaler technique errors associated with asthma outcomes that were not included in the InTeQ because they were specific for MDIs are: ‘incorrect second dose preparation’, ‘poor coordination between the start of the inhalation and the actuation of the dose’, ‘exhaling into the mouthpiece’, and ‘not holding the inhaler upright’. Two generic errors associated with asthma outcomes in the CRITIKAL study which were not included in the InTeQ, ‘not having the head tilted (chin slightly upward)’ and ‘not removing the cap’, merit further research.

In the InTeQ, we chose the approach of patients rating the frequency of correct technique in each step because we prioritized this over the most usual option of measuring whether the patient knows the correct answer or not [19,20]. While being a prerequisite for a good daily inhaler technique, knowing the technique does not necessarily correspond with what patients actually do regularly. Other instruments measure patients’ confidence about their inhaler technique, without considering the actual steps [21,22,23,24,25,26]. Patients’ confidence in the quality of their inhaler technique, in general, may not be a reliable indicator of their actual performance [43] because patients can overestimate their inhaler skills [43,44]. Thus, the focus of the InTeQ on the frequency of performing specific steps may make it a more suitable tool for training and self-monitoring. Repeated instructions on inhaler technique have proven to help achieve effective inhalation skills [45] and improve adherence to therapy and asthma outcomes [11,46].

The Mokken analyses showed that the InTeQ can be considered to be unidimensional and that all the items measure a single underlying construct with good homogeneity. This implies that items can be used to order respondents according to their latent frequency of performing each step, thus justifying the construction of the global score. The high response rate and low proportion of missing values suggest an easy completion for a wide range of patients, thus indicating the feasibility of the InTeQ.

The order of InTeQ items according to the percentage of participants that answered “Always” did not vary among devices in our study, but patients using MDIs answered “Always” to a lower number of steps than patients using other devices. Although the difference was not statistically significant in our study, this pattern is consistent with previous findings [6,7]. In a study that applied the self-reported inhaler technique questionnaire specific for MDIs with 20 questions [20], 68% of the patients reported “exhaling before using their inhaler”, and 77% reported “inhaling slowly and deeply”. In our study, 49% and 75% of the patients using MDIs reported always performing these steps, respectively; the former is probably lower in the InTeQ because of the focus on frequency, which reduces the skewness.

A systematic review of pediatric inhaler technique instruments [17] highlighted the lack of a construct validity assessment (1/24). None of the two prior patient-reported inhaler technique questionnaires assessed this [19,20]. The InTeQ was able to discriminate in the hypothesized direction among known groups based on the support received from healthcare professionals for inhaler technique, indicating the adequate construct validity of the questionnaire. There is evidence supporting the association between a lack of prior inhaler training and poor inhaler technique [47,48]. Although there are several factors associated with poor inhaler technique, such as old age [49], gender [50], a low education level [51], disease-specific knowledge [42], and limited access to primary care [52], we selected inhaler technique training as the most proximally related.

The InTeQ global score can be used for comparing groups, as both Cronbach’s alpha and intraclass correlation coefficients were above the standard of 0.7 [53,54] and reproducibility at item level was moderate [55]. The aforementioned patient-reported questionnaire with 20 items [20] also showed good reliability: Cronbach’s alpha was 0.86 for the total survey score, and 0.94, 0.82, and 0.75 for the subscales. The abovementioned systematic review of pediatric inhaler technique instruments [17] highlighted that reliability was only evaluated in a few studies (4/24), obtaining kappa coefficients for inter-rater agreement from moderate [56] to almost perfect [57,58] and Cronbach’s alpha coefficients from acceptable [59] to excellent [57].

Some potential limitations of this study need to be considered. First, participants may have been affected by social desirability bias when answering the InTeQ. We cannot evaluate criterion validity because the ASTRO-LAB cohort did not include a gold standard measure for inhaler technique performance to limit participant burden. Probably the most suitable gold standard measure for the frequency of correct inhaler technique would be electronic devices added onto the inhalers, but this was found to be unfeasible in this project, and the cost effectiveness of devices is yet to be determined in routine use [60]. Further research should include evaluating the concordance between the InTeQ and objective measurement using electronic devices. Second, since this cohort only included patients up to 40 years old, the results may not be generalized to older adults with asthma. Finally, the items included in the InTeQ are clinically relevant; even so, further work should focus on including all critical steps across inhaler device types, such as the abovementioned two generic errors associated with asthma outcomes in the CRITIKAL study [41], which are not measured in the InTeQ.

## 5. Conclusions

In conclusion, the InTeQ is a feasible, valid, and reliable instrument for self-reporting inhaler technique in any type of device. Patients’ self-monitoring using the InTeQ has the potential to extend the benefit of prior training through the repetition of critical steps, and to help them become aware of under-performing some steps or not performing them as frequently as desired. It can be used by patients to self-monitor their inhaler technique between visits to the healthcare professional, by healthcare professionals to teach patients or other professionals, and by researchers to assess inhaler technique with an easy and accessible tool, which could allow further comparisons among studies.

## Figures and Tables

**Table 1:** InTeQ items and answer options.

Items	Response Options
Breathe out fully before use	Always
Close lips tightly around the mouthpiece	Often
Breathe in deeply through the mouthpiece	Sometimes
Hold my breath for at least 10 s after breathing in	Rarely
Breathe out very slowly after use	Never
	Don’t Know
**Verbatim response regarding options, emerged during a cognitive interview**	
“I think I know how to do it; the doctor asks me to do it in front of them every time I have an asthma clinic. Sometimes I do feel like it is not doing anything, so I do wonder. I take the blue inhaler with me and take a couple of puffs before exercising, and this really worked […] because of actually paying attention on […] how I use it […]. [But] for the morning and evening one, I take it rather quickly, and sometimes I wonder if I shouldn’t actually focus more on how I do it, because sometimes I just take a tiny breath in, and sometimes I am tired or I just woke up and I don’t have any breath capacity, so I do wonder if I do it properly. It’s not because I don’t know, it’s just that it becomes more usual that I use it in a really quick way.”	

**Table 2:** Patients’ characteristics at baseline.

**Primary Care Records**		
Demographic variables		
Age, mean (SD)		28.0 (8.6)
Sex	Male	151 (41.8%)
	Female	210 (58.2%)
Country	France	272 (75.3%)
	United Kingdom	89 (24.7%)
Severity markers 12 months before enrolment		
Asthma-related comorbidities	0	81 (41.1%)
	1	84 (42.6%)
	≥2	32 (16.2%)
	*Missing*	*164 (45.2%)*
Oral corticosteroids courses	0	251 (70.7%)
	≥1	104 (29.3%)
	*Missing*	*6 (1.7%)*
Hospitalizations	No	86 (96.6%)
	Yes	3 (3.4%)
	*Missing*	*272 (75.3%)*
**Telephone interviews at baseline**		
Inhaled controller treatment	Corticosteroids	92 (25.5%)
	Long-acting beta-agonists	14 (3.9%)
	Corticosteroids and long-acting beta-agonists	255 (70.6%)
Inhaler device for controller treatment	Dry powder (DPI)	208 (63.8%)
	Metered-dose (MDI)	61 (18.7%)
	Breath-actuated metered-dose (BA-MDI)	15 (4.6%)
	More than one type of device	42 (12.9%)
	*Missing*	*35 (10.7%)*
Reliever treatment use	Every day	38 (11.4%)
	Almost every day	40 (12.0%)
	Once or twice every week	87 (26.0%)
	Less than once a week	119 (35.6%)
	Never	50 (15.0%)
	*Missing*	27 (7.5%)
Adherence (Medication Intake Survey–Asthma)	Low (≤50%)	81 (27.3%)
	Intermediate (>50–<100%)	87 (29.3%)
	Complete (100%)	129 (43.4%)
	*Missing*	*64 (17.7%)*
**Online survey at baseline**		
Use of spacer during the last 4 months	Always	26 (7.2%)
	Often	13 (3.6%)
	Sometimes	9 (2.5%)
	Rarely	18 (5.0%)
	Never	293 (81.6%)
	*Missing*	*2 (0.6%)*
Make an inhaler use plan together	1 (Not at all)	86 (24.2%)
with healthcare practitioners	2	38 (10.7%)
	3	24 (6.7%)
	4 (In general)	88 (24.7%)
	5	36 (10.1%)
	6	34 (9.6%)
	7 (In a lot of detail)	50 (14.0%)
	*Missing*	*32 (8.2%)*
Healthcare practitioners taught how to use inhalers	1 (Not at all)	85 (23.9%)
	2	24 (6.7%)
	3	20 (5.6%)
	4 (In general)	65 (18.3%)
	5	31 (8.7%)
	6	50 (14.0%)
	7 (In a lot of detail)	81 (22.8%)
	*Missing*	*32 (8.2%)*
**Text messages and telephone interviews**		
Number of exacerbations during the year of follow-up	0	223 (71.9%)
	1	58 (18.7%)
	2	19 (6.1%)
	3–5	10 (3.2%)
	*Missing*	*51 (14.1%)*

Data are presented as *n* (%) or mean (±SD); *n* = 361.

**Table 3:** InTeQ items descriptive statistics, inter-item correlations, and Loevinger’s scalability coefficients.

Item	Mean (SD ^a^)	Skew	Inter-Item Correlations					
			Breathe Out Fully Before	Close Lips Tightly	Breathe In Deeply	Hold Breath After	Breathe Out Slowly	Hi ^b^ (SE ^c^)
**Breathe out fully before**	0.73 (1.12)	1.51	1					0.497 (0.053)
**Close lips tightly**	0.34 (0.67)	2.42	0.33	1				0.566 (0.053)
**Breathe in deeply**	0.23 (0.59)	3.45	0.35	0.55	1			0.708 (0.059)
**Hold breath after**	1.16 (1.30)	0.85	0.32	0.20	0.29	1		0.593 (0.047)
**Breathe out slowly**	1.61 (1.36)	0.37	0.24	0.30	0.24	0.58	1	0.738 (0.045)
**Scale**								**H ^d^ (SE)**
**InTeQ scale**								0.607 (0.040)

^a^ SD: standard deviation; ^b^ Hi: item homogeneity; ^c^ SE: standard error; ^d^ H: scale homogeneity. Mean (SD), skew, and inter-item correlations were estimated from the 5-point Likert scale response options. Homogeneity coefficients with items dichotomized into “Always” vs. the rest, due to their skewed distribution.

**Table 4:** Validity of the InTeQ items and global score comparing known groups defined by the support received from healthcare practitioners and use of spacer.

	Make an Inhaler Use Plan Together ^a^		Teach How to Use the Inhaler		Use of Spacer ^b^	
	1–5: Not Discussed/ Only in General	6–7: Discussed in Detail	1–5: Not Discussed/Only in General	6–7: Discussed in Detail	Always–Sometimes	Rarely–Never
**Subjects**	272	84	225	131	30	68
**Breathe out fully before**						
Always	151 (55.5%)	57 (67.9%)	183 (81.3%)	108 (82.4%)	11 (37.9%)	40 (61.5%)
Often–Sometimes	81 (29.8%)	22 (26.2%)	35 (15.6%)	18 (13.7%)	12 (41.4%)	15 (27.7%)
Rarely–Never	36 (13.2%)	4 (4.8%)	6 (2.7%)	2 (1.5%)	6 (20.7%)	7 (10.8%)
Don’t know	3 (1.1%)	1 (1.2%)	1 (0.4%)	2 (1.5%)	0 (0.0%)	0 (0.0%)
*p*-value	**0.049**		0.246		0.097	
**Close lips tightly**						
Always	127 (56.4%)	81 (61.8%)	110 (40.4%)	45 (53.6%)	21 (70.0%)	49 (72.1%)
Often–Sometimes	65 (28.9%)	38 (29.0%)	102 (37.5%)	27 (32.1%)	9 (30.0%)	18 (26.5%)
Rarely–Never	30 (13.3%)	10 (7.6%)	52 (19.1%)	12 (14.3%)	0 (0.0%)	1 (1.5%)
Don’t know	3 (1.3%)	1 (0.8%)	3 (1.1%)	0 (0.0%)	0 (0.0%)	0 (0.0%)
*p*-value	0.108		0.585		0.760	
**Breathe in deeply**						
Always	191 (70.2%)	68 (81.0%)	92 (40.9%)	63 (48.1%)	19 (63.3%)	56 (82.4%)
Often–Sometimes	66 (24.3%)	16 (19.0%)	84 (37.3%)	45 (34.4%)	8 (26.7%)	11 (16.2%)
Rarely–Never	6 (2.2%)	0 (0.0%)	44 (19.6%)	20 (15.3%)	2 (6.7%)	0 (0.0%)
Don’t know	8 (2.9%)	0 (0.0%)	1 (0.4%)	2 (1.5%)	1 (3.3%)	1 (1.5%)
*p*-value	0.227		0.507		0.070	
**Hold breath after**						
Always	162 (72.0%)	97 (74.0%)	69 (25.4%)	28 (33.3%)	8 (26.7%)	37 (54.4%)
Often–Sometimes	54 (24.0%)	28 (21.4%)	114 (41.9%)	36 (42.9%)	13 (43.3%)	22 (32.4%)
Rarely–Never	5 (2.2%)	1 (0.8%)	77 (28.3%)	18 (21.4%)	9 (30.0%)	8 (11.8%)
Don’t know	4 (1.8%)	4 (3.1%)	3 (1.1%)	0 (0.0%)	0 (0.0%)	1 (1.5%)
*p*-value	0.176		0.381		**0.035**	
**Breathe out slowly**						
Always	216 (79.4%)	75 (89.3%)	55 (24.4%)	42 (32.1%)	5 (16.7%)	23 (34.4%)
Often–Sometimes	45 (16.5%)	8 (9.5%)	96 (42.7%)	54 (41.2%)	23 (50.0%)	31 (46.3%)
Rarely–Never	7 (2.6%)	1 (1.2%)	66 (29.3%)	29 (22.1%)	10 (33.3%)	12 (17.9%)
Don’t know	3 (1.1%)	0 (0.0%)	1 (0.4%)	2 (1.5%)	0 (0.0%)	1 (1.5%)
*p*-value	0.333		0.355		0.178	
**Quality of inhalation technique according to InTeQ global score**						
Poor (0–2 “Always”)	114 (41.9%)	22 (26.2%)	91 (40.1%)	45 (34.4%)	17 (56.7%)	24 (35.3%)
Fair (3 “Always”)	71 (26.1%)	24 (28.6%)	62 (27.6%)	33 (25.2%)	6 (20.0%)	16 (23.5%)
Good (4–5 “Always”)	87 (32.0%)	38 (45.2%)	72 (32.0%)	53 (40.5%)	7 (23.3%)	28 (41.2%)
*p*-value	**0.023**		0.264		0.120	

^a^ Make a concrete plan together of where, when, and how to use the inhalers; ^b^ Patients using MDI; *p*-value in bold type indicates *p* < 0.05.

**Table 5:** Reproducibility of the InTeQ evaluated among stable participants at baseline and 12 months.

		12 Months		% of Agreement (95%CI ^a^)	Kappa (SE ^b^)
**InTeQ Item**	**Baseline**	**Always**	**Often–Never**		
**Breathe out fully before**	**Always**	36	6	73.8 (68.8–78.7)	0.468 (0.097)
	**Often–Never**	15	23		
**Close lips tightly**	**Always**	51	9	78.8 (74.2–83.3)	0.443 (0.114)
	**Often–Never**	8	12		
**Breathe in deeply**	**Always**	60	11	80.0 (75.5–84.5)	0.224 (0.135)
	**Often–Never**	5	4		
**Hold breath after**	**Always**	27	10	78.8 (74.2–83.3)	0.570 (0.092)
	**Often–Never**	7	36		
**Breathe out slowly**	**Always**	15	5	81.3 (76.8–85.8)	0.551 (0.105)
	**Often–Never**	9	46		
**InTeQ global score**	**Mean (SD ^c^)** **baseline**	**Mean (SD ^c^)** **12 m**	**Mean (SD ^c^)** **change**	***p*-value**	**ICC ^d^**
	2.8 (1.5)	2.9 (1.7)	0.06 (1.34)	0.769	0.775

^a^ 95%CI: confidence interval; ^b^ SE: standard error; ^c^ SD: standard deviation; ^d^ ICC: intraclass correlation coefficient; *n* = 81.

## Data Availability

The data presented in this study are available on request from the corresponding author.
